# Point-of-care testing in UK primary care: a survey to establish clinical needs

**DOI:** 10.1093/fampra/cmw018

**Published:** 2016-04-05

**Authors:** Philip J Turner, Ann Van den Bruel, Caroline H D Jones, Annette Plüddemann, Carl Heneghan, Matthew J Thompson, Christopher P Price, Jeremy Howick

**Affiliations:** ^a^Nuffield Department of Primary Care Health Sciences, University of Oxford, Oxford, UK and; ^b^Department of Family Medicine, University of Washington, Seattle, WA, USA.

**Keywords:** Cross-sectional studies, diagnostic tests, general practitioners, point-of-care, primary health care, surveys and questionnaires.

## Abstract

**Background.:**

A number of point-of-care diagnostic tests are commercially available in the UK, however, not much is known regarding GPs’ desire for these tests or the clinical areas of interest.

**Objective.:**

We sought to establish for which conditions point-of-care tests (POCTs) would be most helpful to UK GPs for diagnosis, reduction of referrals, and monitoring of chronic conditions.

**Methods.:**

A total of 1635 regionally representative GPs were invited to complete an online cross-sectional survey between 31 September and 16 October 2012.

**Results.:**

A total of 1109 (68%) GPs responded to the survey. The most frequently cited conditions were urinary tract infections for diagnosis (47% of respondents), pulmonary embolism/deep vein thrombosis for referral reduction (47%) and international normalized ratio/anticoagulation for monitoring (49%).

**Conclusions.:**

This survey has identified the conditions for which UK GPs would find POCTs most helpful. Comments by respondents suggest that quite radical system-level adjustments will be required to allow primary care clinicians to capitalize on the potential benefits of POCTs.

## Introduction

The last decade has seen an increasing focus on delivering health care closer to home and a more integrated approach to care delivery (1,2). Supporting care closer to home improves patient satisfaction, improves access and has been shown to be safe and clinically effective, although cost effectiveness has not yet been proven (3). This trend has been complemented by a call for newer models of care (4), driven by the need for more patient-centred care, reduction of unplanned admissions, better care for patients with long-term conditions and cost containment. One way of achieving care closer to home is implementing point-of-care tests (POCTs) during a single practice visit in the assumption that this might reduce the need for additional testing elsewhere, repeat visits or referrals caused by diagnostic uncertainty; however, practice-based diagnostic services are not commonly available to GPs in the UK (5).

This trend is being accompanied by a move in commissioning of services from activity to outcome based (6). Consequently, work should focus on how test results improve clinical decision-making, patient management, referrals (urgent and non-urgent) and care process efficiency. A recent systematic review showed that although clinicians considered that POCTs could aid decision-making and management of conditions, there were concerns associated with test accuracy, clinicians becoming overly reliant on tests, cost and limited utility (7). Further studies have also highlighted concerns about access to tests in primary care, the choice of tests and the interpretation of results (8–10).

There have, however, been very few attempts to determine the tests that clinicians would require in primary care (11,12). New diagnostic technologies are generally prioritized by clinicians based on their potential impact on mortality and morbidity, their diagnostic accuracy and potential to improve the delivery of care (11). For a number of years, though, the evidence base for diagnostic services has remained limited with a heavy emphasis on technical assessment (13).

Nevertheless, establishing a clinical need is a vital step in technology development, making successful adoption more likely. To address this, we aimed to identify the POCTs that primary care GPs require access to and would consider using in practice, focussing on blood, urine and other biological fluids that usually require laboratory investigations.

## Methods

We conducted an online cross-sectional survey of GPs in the UK. The survey was developed using an established survey tool and included questions on the impact of POCTs on decision-making by GPs and in what clinical scenarios POCTs could be of value. The survey focussed on three clinical scenarios (i) diagnosis (rule-in or rule-out), (ii) reduction of referrals and (iii) monitoring and management of conditions. The initial survey was developed by a group of clinicians and clinical scientists (JH, CJ, MT, AVdB and CPP) based on laboratory test usage frequency in Oxfordshire; the survey was subsequently pilot tested with 30 GPs and modified based on feedback received from the pilot survey cohort (14) (see online Supplementary Appendix 1 for the full survey). One part of the survey, i.e. the POCTs related to clinical scenarios on ‘diagnosis’, was subsequently adapted for use in an international survey of primary care physicians, the results of which were published (14). We report here the detailed results of the survey including POCTs related to all three scenarios, i.e. diagnosis, referrals and monitoring, in UK respondents.

### Implementation of survey

The survey was distributed by Doctors.net, which hosts an online survey tool and holds registrations of 71% of UK GPs. An invitation was sent to 1635 regionally representative GPs, sampled from among the UK GP membership, via email on 31 September 2012, and the survey closed on 16 October 2012. GPs were sampled by the allocation of quotas by nation/region of the UK, with the cohort randomly selected within each regional quota. Invitations to complete the survey were also displayed on the Doctors.net homepages of selected participants.

We asked respondents in which conditions/illnesses they felt that POCTs would be most useful for (i) diagnosis, (ii) reducing referrals and (iii) monitoring and management (questions 1, 3 and 2, respectively, of the survey; Supplementary Appendix 1); respondents could list up to five conditions in each of these three categories, regardless of whether there was a POCT currently available. We asked respondents to share any comments they had including benefits and concerns about POCTs. We collected demographic information on gender, year of qualification as a doctor, number of hours worked per week, their role in their practice (e.g. partner, salaried and locum) and number of patients registered in the practice. In addition, we asked about the distance from the practice to the nearest emergency department that admitted patients to hospital, the length of time to get the results of a routine blood test from the laboratory and whether the practice location was urban, suburban, semi-rural or rural.

### Data analysis

We categorized the cited conditions according to the International Classification of Primary Care (ICPC-2-R) system (15). When there was no code in the ICPC system for a specific condition (e.g. cancer), an additional unique code was created. Some of the categories had to be combined; for example, in some responses pulmonary embolism (PE) and deep vein thrombosis (DVT) were listed as separate conditions, while in others they were listed as one combined condition; subsequently, we combined PE and DVT in a single category. Four authors modified the ICPC codes through discussion (PJT, JH, MT and AVdB). The full details of the modified ICPC-2-R can be found in Supplementary Table 1.

Data were subsequently extracted to SPSS version 22 for all statistical analyses; descriptive statistics were calculated, and recorded conditions were coded and frequencies determined. We examined whether time taken to receive blood test results, distance from the nearest hospital with an emergency department and practice size were associated with the number of conditions listed, as a proxy for willingness to implement POCTs. We assigned respondent demographic data to six groups based on number of conditions recorded (0–5) for each category of testing. We used the non-parametric Kruskal–Wallis *H*-test, independent one-way analysis of variance on ranks to compare across groups as group data were not normally distributed and group sizes differed widely.

All 818 free-text responses (Q6 of the survey) regarding benefits and concerns associated with POCTs were initially cleaned to remove non-valid entries, e.g. ‘n/a’, ‘no comment’ or similar (195 such entries were eliminated). We then examined responses and developed a list of codes based on their content. Responses were allocated to codes: comments could be assigned to multiple codes as appropriate. Finally, we collated the codes into three main themes of facilitators and barriers to adoption of POCTs: clinician level, patient level and system level.

## Results

### Characteristics of respondents

A total of 1109 UK GPs responded (68% response rate) (Table 1). Female clinicians (43%) were slightly under-represented when compared with national data for 2012 [England 47% (16)] and practices were on average larger [8275 patients; England average 6891 (16)].

**Table 1.** Characteristics of GPs from the UK who responded to the Doctors.net-hosted online survey between 31 September and 16 October 2012

Total number of respondents	1109
Male (%)	634 (57)
Female (%)	475 (43)
Year of qualification, median	1996 (range 1961–2009)
Miles to nearest hospital, median	5 (range 0–100)
Time to blood results, days, median	1.0 (range 0–24)
Location of practice	
Rural or semi-rural (%)	377 (34)
Urban or suburban (%)	732 (66)
Number of patients registered to practice, mean	8275 (SE 122)
Source	Doctors.net
Type of survey	Electronic
Dates of data collection	Sent out September 2012, closed October 2012

### Conditions for which point-of-care tests would be helpful to UK GPs

For diagnosis, 1082 respondents (98%) listed 4195 discreet entries covering 107 conditions (median 4 conditions per respondent; range 0–5) for which they felt POCTs would help them in their diagnostic decision-making. Table 2 shows the top three were urinary tract infections (UTI), pulmonary embolism/deep vein thrombosis (PE/DVT) and diabetes (not otherwise specified). For reducing referrals, 81% (902) respondents listed 2416 entries covering 104 conditions (median 2 conditions per respondent; range 0–5) for which POCTs could help. Table 3 shows the top three were PE/DVT, acute cardiac disease and diabetes (not otherwise specified). Finally, 1042 (94%) of respondents recorded 3285 entries in 92 different conditions (median 3; range 0–5) for which POCTs could assist with monitoring of long-term conditions, with the top three being international normalized ratio (INR)/anticoagulation, diabetes (not otherwise specified) and acute and chronic renal impairment/failure (Table 4).

**Table 2.** Conditions for which respondents considered that point-of-care tests would help them with diagnosis: top 20 in the UK

Diagnosis		
Number of respondentsRespondents reporting conditionsNumber of conditions recorded	110910824195	
Condition	Percentage of total recorded conditions (*n*)	Percentage of respondents recording condition
Urinary tract infection	12.4 (521)	47.0
Pulmonary embolism/deep vein thrombosis	11.4 (478)	43.1
Diabetes (not otherwise specified)	9.2 (387)	34.9
Acute cardiac disease	6.7 (282)	25.4
International normalized ratio/anticoagulation	4.7 (199)	17.9
Pregnancy	4.2 (178)	16.1
Anaemia	3.9 (162)	14.6
Heart failure	3.0 (124)	11.2
Chronic obstructive pulmonary disease/asthma	2.8 (116)	10.5
Chest infection/cough/lower respiratory tract infection	2.4 (102)	9.2
Diabetes (glucose)	2.3 (98)	8.8
Lipid disorder	2.2 (92)	8.3
Strep throat/tonsillitis	2.0 (85)	7.7
Cancer	2.0 (85)	7.7
Sexually transmitted diseases	2.0 (84)	7.6
Acute and chronic renal impairment/failure	2.0 (84)	7.6
Human immunodeficiency virus/acquired immunodeficiency syndrome	1.3 (55)	5.0
Hyper/hypothyroidism	1.3 (54)	4.9
Cardiovascular disease, other	1.2 (49)	4.4
Acute infection (bacterial versus viral not otherwise specified)	1.0 (40)	3.6

**Table 3.** Conditions for which respondents considered that point-of-care tests would help them to reduce referrals: top 20 in the UK

Referrals		
Number of respondentsRespondents reporting conditionsNumber of conditions recorded	11099022416	
Condition	Percentage of total recorded conditions (*n*)	Percentage of respondents recording condition
Pulmonary embolism/deep vein thrombosis	21.4 (517)	46.6
Acute cardiac disease	11.2 (271)	24.4
Diabetes (not otherwise specified)	5.5 (133)	12.0
Chronic obstructive pulmonary disease/asthma	5.0 (122)	11.0
Heart failure	4.8 (116)	10.5
International normalized ratio/anticoagulation	4.1 (100)	9.0
Urinary tract infection	3.1 (74)	6.7
Cancer	2.9 (70)	6.3
Acute and chronic renal impairment/failure	2.6 (64)	5.8
Chest infection/cough/lower respiratory tract infection	2.0 (48)	4.3
Anaemia	1.6 (39)	3.5
Ectopic pregnancy	1.5 (37)	3.3
Musculoskeletal inflammation (including rheumatic disease)	1.4 (35)	3.2
Acute infection (bacterial versus viral not otherwise specified)	1.3 (32)	2.9
Abdominal pain	1.2 (29)	2.6
Cardiovascular disease, other	1.1 (27)	2.4
Pregnancy	1.1 (26)	2.3
Peptic ulcer	1.0 (23)	2.1
Urea and electrolytes	0.8 (20)	1.8
Appendicitis	0.7 (18)	1.6

**Table 4.** Conditions for which respondents considered that a point-of-care test would help them to monitor or manage patients’ conditions: top 20 in the UK

Monitoring		
Number of respondentsRespondents reporting conditionsNumber of conditions recorded	110910423285	
Condition	Percentage of total recorded conditions (*n*)	Percentage of respondents recording condition
International normalized ratio/anticoagulation	16.7 (547)	49.3
Diabetes (not otherwise specified)	16.0 (527)	47.5
Acute and chronic renal impairment/failure	7.0 (230)	20.7
Chronic obstructive pulmonary disease/asthma	6.8 (223)	20.1
Lipid disorder	4.7 (154)	13.9
Hyper/hypothyroidism	3.8 (126)	11.4
Anaemia	3.7 (121)	10.9
Musculoskeletal inflammation (including rheumatic disease)	3.2 (105)	9.5
Pulmonary embolism/deep vein thrombosis	3.2 (104)	9.4
Cancer	3.0 (100)	9.0
Heart failure	2.8 (93)	8.4
Urinary tract infection	2.5 (83)	7.5
Diabetes insulin dependent/diabetes, non-insulin dependent (HbA1c testing)	2.2 (73)	6.6
Acute cardiac disease	1.5 (49)	4.4
Cardiovascular disease, other	1.5 (49)	4.4
Hypertension	1.2 (41)	3.7
Diabetes (glucose)	1.1 (37)	3.3
Acute infection (bacterial versus viral not otherwise specified)	1.0 (34)	3.1
Chest infection/cough/lower respiratory tract infection	1.0 (32)	2.9
Rheumatoid arthritis/osteoarthritis drug monitoring	0.9 (29)	2.7

The complexity of diabetes was acknowledged in the modified ICPC-2 coding used (Supplementary Table 1), although only the most frequently recorded codes appeared in the top 20 Tables 2–4. A full breakdown of diabetes-related entries recorded by respondents appears in Supplementary Table 2 for completeness. Although not coded in the ICPC-2, we subdivided the Cancer (all) code and present these data in Supplementary Table 3. The most dominant cancer types recorded were urological cancers, with cancer of the prostate being the dominant cancer in this subgroup (72%, 85% and 95% of urological cancer entries for diagnosis, referral reduction and monitoring and management, respectively).

### Potential correlations between demographic data and number of conditions listed by respondents

We could not identify any significant relationships between demographic factors (time to receive blood test results, distance of practice from nearest emergency department and practice size) and the numbers of conditions recorded for which respondents considered that POCTs would be helpful for any of the three clinical scenarios (See Supplementary Table 4).

### Free-text responses

A total of 623 free-text responses were valid entries (by 56% of the 1109 respondents). Emergent themes, separated into perceived facilitators and barriers to implementation of POCTs, are summarized in Table 5.

**Table 5.** Summary of UK GPs’ attitudes towards point-of-care tests and how these may drive or inhibit adoption in primary care

	Facilitators	Barriers
Clinician level		
Easier access	Assist with diagnosis	Erosion clinician’s diagnostic capabilities
		Excessive testing
		Excessive patient demand for tests
	Improved job satisfaction	
Immediate result	Rapid decision-making	Eliminates time for watchful waiting
	Targeted prescribing	Increase in prescribing
	Reduction of referrals	Increase in referrals
		Immediate results driving demand for immediate action
		Will take more GP time
Accuracy		Concerns on accuracy and reliability
Patient level		
Easier access	Improved satisfaction because better convenience	
Immediate result	Correct patient receives result	
System level		
Easier access	Remote practices can improve care	
Clinical governance		Requirement of clinical governance structures to be set-up
		Need for quality control, calibration and maintenance
		Legal liability: who would be responsible?
Costs		Drain on practice budgets
		Costs prohibitive for small rural practices
		Time and costs associated with quality control
Expertise		Additional training requirements for staff

#### At the clinician level

Some respondents entered generally positive or negative comments about POCTs, but did not qualify their entries further. A number of respondents explained that POCTs would assist them because they make access to testing easier: ‘I think that some benefits are very great—including more targeted care…’; however, others expressed concerns tests would erode clinicians’ capability to make clinical judgements: ‘Increased access to tests decreasing clinical acumen and judgement’.

Some clinicians felt availability of tests may result in excessive testing of patients, driven by doctors as well as patient demand: ‘May lead to over-testing’; ‘Over testing likely’.

Some respondents felt POCTs would enable faster decisions ‘Quick results enable fast decisions’. However, others expressed concern related to their workload and the current structure of appointments ‘Concerns; will take more GP time – there is NO spare time left’.

Positive comments were recorded concerning the potential benefit of POCTs for appropriate prescribing: ‘more targeted care for e.g. throat infections and a good way to reduce antibiotic therapy where it is not required’. Potential negative impacts on prescriptions were also mentioned ‘.there is every possibility that the patient might get better without treatment, the positive test makes you treat the condition…’. While some respondents felt POCTs would reduce referral to secondary care, others expressed concerns: ‘No point doing a test if you do not know how to interpret it and explain it, need specialist knowledge, may increase referrals otherwise’.

Respondents recorded concerns regarding the accuracy of results from POCTs: ‘Concerns about accuracy in comparison with proper blood tests especially for diagnostic purposes’.

#### At the patient level

Some doctors considered POCTs would improve patient experience: ‘availability of POCTs would increase patient satisfaction markedly—patients dislike having to book separate appointments to have a test done, then come back for results etc’.

#### System level

Respondents who stated that their practices were remote or isolated suggested that POCTs would be helpful. The need for training and calibration and maintenance of equipment were also noted. Concerns about cost and funding for POCT testing were evident: ‘There is no sensible way of reimbursing the cost of these tests at present’.

Concerns were expressed regarding legal responsibility and risk to the user associated with POCTs: ‘Potential legal ramifications of choosing NOT to use them’, ‘Main concerns would be around sensitivity/specificity and medico-legal implications…’. Some respondents questioned how results from POCTs might be transferred to and integrated with the clinical records system. There was also concern that POCT use would result in loss of laboratory facilities.

## Discussion

This survey has identified the range of conditions for which UK GPs consider that POCTs could assist with diagnosis, reduction of referral and monitoring and management. Conditions most commonly cited were UTI for diagnosis, PE/DVT for reducing referrals and INR/anticoagulation for monitoring and management. Almost half of respondents considered that POCTs for each of these conditions would be helpful. Considerable overlap also existed between conditions and the categories of use suggesting that some diagnostic tests have potential utility across the spectrum of diagnosis and patient management.

Conditions listed in the top 10 for all 3 categories were PE/DVT, diabetes, INR/anticoagulation and chronic obstructive pulmonary disease (COPD)/asthma. For diabetes, respondents listed several aspects of diagnosis and monitoring (e.g. HbA1c, blood glucose, urine tests for glucose and creatinine) as well as associated conditions (e.g. diabetic ketoacidosis)—the coding reflects this complexity. When all these aspects of the disease are considered together, diabetes is clearly a very prominent condition for which GPs would like to use POCT.

Respondents listed a wide range of conditions for which they felt that POCTs would assist with the three testing scenarios. This could reflect variability in the respondents’ patient populations and local incidence/prevalence of conditions. It could also be an indication of variability in the clinical backgrounds and training of respondents influencing the conditions they listed. Additionally, the wide range recorded could simply reflect the fact that there are many conditions for which POCTs might be useful.

In the case of better diagnosis, there appear to be two categories: common but not very serious conditions such as UTI, which might have immediate treatment indications such as antibiotic prescription, and rare and potentially very serious conditions that require timely diagnosis and referral such as PE/DVT. UTI is a common condition for which a POCT could potentially change practice. Currently available tests for UTI are either only moderately accurate (dipstick tests) or provide results too late to influence immediate clinical decision-making (urine culture). As a result, most patients with suspected UTI are managed empirically, leading to suboptimal use of antibiotics and downstream consequences of antimicrobial resistance. An observational study in UK general practice found that 60% of women prescribed empirical antibiotics for suspected UTI were found to be culture negative for bacterial infection and 25% of women not prescribed antibiotics were positive for bacterial infection (17). PE and DVT are associated with a considerable risk of mortality, with 30% of patients dying within 30 days and of those who survive, 28% develop venous stasis syndrome (18). Primary care clinicians are tasked with timely recognition and referral for prompt treatment while at the same time avoiding unnecessary referrals since they may swamp secondary care services that are already stretched.

The fact that INR/anticoagulation was recorded by many respondents as a POCT that they would want to use in the future despite the fact that several tests are already currently commercially available suggests that either the currently available tests do not meet the essential requirements for successful adoption or that the system is currently blocking adoption perhaps because of lack of governance processes, quality assurance or procurement/financial issues.

A number of the facilitators and barriers to POCT testing that emerged from this survey were similar to those identified by a systematic review of qualitative studies that examined clinicians’ attitudes towards POC blood testing (7). The common facilitators were increased diagnostic certainty, more accurate prescription of treatment, fewer re-consultations/referrals and improved patient satisfaction. Common barriers included concerns regarding diagnostic accuracy, impact of testing on clinical skills, costs associated with use and maintenance and added pressure on clinician time. Although the comments suggest that there is desire for POCTs among some UK GPs, considerable barriers to uptake prevail at the clinician, patient and system levels. Many of the barriers can ultimately be traced back to system-level issues related to workload, models of reimbursement, together with governance and legal support. It is clear that quite radical system-level changes will be required to enable clinicians to take advantage of the potential benefits of POCTs in UK primary care.

### Limitations

For most of the top conditions listed, POCTs are currently commercially available in the UK. A weakness of the survey was that it did not explicitly ask clinicians to quantitatively indicate their desire for tests, but asked them to list conditions for which POCTs would be most useful for each category. Hence it is not possible to comment directly on unmet need from the ranking data presented in Tables 2–4, however, these data do provide an indication of the conditions in which rapid access to results would be important in decision-making for UK GPs. The formulation of the referrals question was problematic, as it asked respondents to record conditions for which POCTs could reduce referrals. It would have been preferable to emphasize guidance of referral rather than reduction, as the original question was narrow and potentially missed conditions where diagnostics could assist clinicians to refer most appropriately.

Another limitation of our survey is that we can not estimate how frequently tests would be used for certain conditions. For example, the majority of our respondents indicated they would want to have a POCT for PE/DVT. However, it is fair to assume that if in place, such a POCT would not be used daily as these presentations are not very common.

Some of the open-text responses to questions were either ambiguous or listed multiple conditions in a single field. It was thus not possible for us to accurately categorize all condition entries, with 3.8%, 5.7% and 3.6% of all responses unclassifiable for diagnosis, referral reduction and monitoring, respectively. This is an inherent issue with questions that require free-text responses, and it is difficult to see how this could be avoided.

The survey was restricted to GPs and did not include other primary care professionals, e.g. nurses and midwives, who are also involved in monitoring and management of patients’ conditions. A comprehensive assessment of need in primary care would require input from all clinical stakeholders.

## Conclusions

This is the first survey of the needs of clinicians, in primary care, for the rapid delivery of results using POC testing that has approached the issue from the range of potential clinical utilities linked to clinical decision-making, as against the choice of the test. This work highlights conditions for which POCTs could aid diagnosis, reduce referrals, aid monitoring and management of conditions, and indicates some of the barriers to adoption. For many of the conditions listed by the respondents, POCTs are already available but not utilized. Earlier studies have shown that barriers include accuracy concerns, perceived lack of capacity to alter consultations, potentially misleading results, limited usefulness and patient anxiety resulting from intermediate results (7). Implementation research evaluating real-life benefits and barriers could help in improving the match between clinical needs and technological possibilities. For other conditions, there is currently a paucity of good POCTs available that address the real issues. This is the case for UTIs where current POCTs are not sufficiently accurate to improve efficiency in antibiotic prescribing. Both implementation research and identification of areas where current POCTs are suboptimal may be worthy foci for future studies.

## Supplementary material

Supplementary material is available at *Family Practice* online.

## Declarations

Funding: This work was funded in part by educational grants from the following companies: Alere, Atlas Genetics, BD (Becton Dickinson and Company), Ortho Clinical Diagnostics, Philips Home Clinical Monitoring (the Netherlands), Siemens Healthcare Diagnostics, and Nova Biomedical. PJT, AVdB, AP, CHDJ, CH, CPP are supported through the National Institute for Health Research (NIHR) Diagnostic Evidence Co-operative Oxford at Oxford Health Foundation Trust (award number IS_DEC_0812_100) (14). The study sponsors had no role in the design, analyses or reporting of the study. The authors retained complete independence in the conduct of this work.

Ethical approval: none.

Conflict of interest: MT has received funds for research related to point-of-care tests from Alere Inc, Coulter Foundation, and Roche Molecular Diagnostics. AVdB is an associate editor of *Family Practice*.

## Supplementary Material

Supplementary Data
