# Supplementary material for: Point-of-care testing in UK primary care: a survey to establish clinical needs
Source: Fam Pract. 2016 Apr 5;33(4):388–94. doi: 10.1093/fampra/cmw018 (PMC4957010; doi:10.1093/fampra/cmw018)
Supplement: Supplementary Data [file supp_33_4_388__index.html]

Point-of-care testing in UK primary care: a survey to establish clinical needs — Point-of-care testing in UK primary care: a survey to establish clinical needs — Supplementary Data 

# Point-of-care testing in UK primary care: a survey to establish clinical needs

## Supplementary Data

Data files

- Supplementary Data - Supplementary Data
