# Supplementary material for: Point-of-care testing in UK primary care: a survey to establish clinical needs
Source: Fam Pract. 2016 Apr 5;33(4):388–94. doi: 10.1093/fampra/cmw018 (PMC4957010; doi:10.1093/fampra/cmw018)
Supplement: Supplementary Data [file supp_cmw018_UK_POC_needs_supplementary_revs.docx]

# Supplementary information

# Supplementary Appendix 1: Full Survey (UK version)

**POCT (Point of care tests) study**

Doctors.net.uk invites you to participate in a survey commissioned by an academic institution concerning usage of Point of Care Tests. The survey will take around 5 minutes to complete. All eligible members completing the survey will receive 1,000 eSR points. Please read the following text, which explains the intent of this research.

Doctors.net.uk would like to reassure you that:

- Doctors.net.uk will comply with all UK laws protecting your personal data and the British Healthcare Business Intelligence Association and Market Research Society guidelines
- Your responses will be used by us and the sponsoring academic institution for market research only. All information included is for research only.
- Your responses will be collated with other respondents and presented to the sponsor in aggregated or anonymised form
- Your responses will be confidential and will not be used for any other purposes or disclosed to any third party without your approval.

Please confirm that you have read and understood this information

Yes

No *CLOSE*

We would like to know about your use of, and opinions about, tests that could be delivered quickly in your practice – namely **Point of Care Tests (POCTs), which are also known as ‘near-patient tests’.**

By **Point of Care Tests (POCTs)** we mean tests that are done in a primary care setting with results becoming available during the clinic visit. We are asking you about POCTs on samples taken from the body, including blood, urine and other bodily fluids.

You will be familiar with some tests, and others will be unknown to you (and new POCTs are always being developed). We think it is important to find out which tests GPs use and would like to use.

**You will be able to view this definition again later in the survey by mousing over “Point of Care Tests (POCTs),” in the text of questions that concern them.**

If you would like any more information about this project then please contact Dr Jeremy Howick or Dr Caroline Jones at the Department of Primary Care Health Sciences, Oxford (Jeremy.howick@phc.ox.ac.uk; Caroline.jones@phc.ox.ac.uk).

Are you happy to proceed with the interview on this basis?

Yes

No *CLOSE*

Point of care tests are designed to give clinicians a rapid result to a test using blood, urine, respiratory samples or other body fluids. We would like you to tell us **in** **which CONDITIONS / ILLNESSES you feel that point of care tests (POCTs) would be most useful, in different situations** (diagnosis, monitoring, and reducing referrals).

Q1 Diagnosis

Please name up to 5 conditions for which a POCT could help you make a **DIAGNOSIS**. Please list the conditions irrespective of whether or not POCTS currently exist

1. _____________  (please specify)
2. _____________  (please specify)
3. _____________  (please specify)
4. _____________  (please specify)
5. _____________  (please specify)

- I do not believe POCTS would help me make a diagnosis

*Open end; Must select “Open End a” or ”I do not believe…” ; Open ends b-e are non-mandatory*

Q2 Monitoring

Please name up to 5 conditions that a POCT could help you **MONITOR** or manage. Please list the conditions irrespective of whether or not POCTS currently exist

1. _____________  (please specify)
2. _____________  (please specify)
3. _____________  (please specify)
4. _____________  (please specify)
5. _____________  (please specify)

- I do not believe POCTS would help me monitor or manage conditions

*Open end; Must select “Open End a” or ”I do not believe…” ; Open ends b-e are non-mandatory*

Q3 Reduction of referrals

Please name up to 5 conditions for which a POCT could help you **REDUCE REFERRALS for specialty care or hospital admission**. Please list the conditions irrespective of whether or not POCTS currently exist

1. _____________  (please specify)
2. _____________  (please specify)
3. _____________  (please specify)
4. _____________  (please specify)
5. _____________  (please specify)

- I do not believe POCTS would help me make a diagnosis

*Open end; Must select “Open End a” or ”I do not believe…” ; Open ends b-e are non-mandatory*

Q4 POCTs used

**Please select the answer that best matches your views about current or potential use of point of care tests (POCTs)**

*We are aware that this is a long list but this data is critical to the study and this is the longest question.*

|  | This test **is** currently available as a point of care test (POCT) in my clinic | | This test **is not** currently available as a point of care test (POCT) in my clinic | |
| --- | --- | --- | --- | --- |
|  | *(1)* I **do** use this test | *(2)* I **do not** use this test | *(3)* I **would** use this test | *(4)* I **would not** use this test |
| **TESTS ON BLOOD** |  |  |  |  |
| **Cardiovascular** |  |  |  |  |
| Creatinine |  |  |  |  |
| Potassium |  |  |  |  |
| Sodium |  |  |  |  |
| Total cholesterol |  |  |  |  |
| HDL/LDL cholesterols |  |  |  |  |
| Triglycerides |  |  |  |  |
| Calcium |  |  |  |  |
| Uric Acid |  |  |  |  |
| BNP (B-natriuretic peptide) |  |  |  |  |
| D-dimer |  |  |  |  |
| Troponin |  |  |  |  |
| **Endocrine** |  |  |  |  |
| Blood glucose |  |  |  |  |
| HbA1c |  |  |  |  |
| TSH (thyroid stimulating hormone) |  |  |  |  |
| Free T4 or T3 |  |  |  |  |
| **Haematology** |  |  |  |  |
| INR |  |  |  |  |
| Haemoglobin |  |  |  |  |
| White cell count |  |  |  |  |
| Platelet count |  |  |  |  |
| Prothrombin time |  |  |  |  |
| **Infection related** |  |  |  |  |
| CRP (C-reactive protein) |  |  |  |  |
| Procalcitonin |  |  |  |  |
| HIV blood test |  |  |  |  |
| Hepatitis B |  |  |  |  |
| **Liver** |  |  |  |  |
| AST/ALT |  |  |  |  |
| Alkaline phosphatase |  |  |  |  |
| Bilirubin |  |  |  |  |
| Gamma GT (ɣ-glutamyltransferase) |  |  |  |  |
| Albumin |  |  |  |  |
| **Other (blood)** |  |  |  |  |
| ESR (Erythrocyte sedimentation rate) |  |  |  |  |
| CA125 |  |  |  |  |
| PSA (Prostate Specific Antigen) |  |  |  |  |
| Vitamin D |  |  |  |  |
| Vitamin B12 |  |  |  |  |
| Folate |  |  |  |  |
| Quantitative Beta HCG (Human chorionic gonadotropin) |  |  |  |  |
| Rheumatoid factor |  |  |  |  |
| ANA (anti-nuclear antibodies) |  |  |  |  |

|  | This test **is** currently available as a point of care test (POCT) in my clinic | | This test **is not** currently available as a point of care test (POCT) in my clinic | |
| --- | --- | --- | --- | --- |
|  | *(1)* I **do** use this test | *(2)* I **do not** use this test | *(3)* I **would** use this test | *(4)* I **would not** use this test |
| **RESPIRATORY SAMPLES** |  |  |  |  |
| Throat swab for Group A Streptococci |  |  |  |  |
| Nasal swab for MRSA |  |  |  |  |
| Nose/throat swab for influenza |  |  |  |  |
| **TESTS ON URINE OR GENITAL FLUIDS** |  |  |  |  |
| Urine pregnancy test |  |  |  |  |
| Urine leucocytes or nitrite |  |  |  |  |
| Chlamydia |  |  |  |  |
| Gonorrhoea |  |  |  |  |
| Urine albumin:creatinine ratio |  |  |  |  |
| Urine total protein |  |  |  |  |
| Urine protein:creatinine ratio |  |  |  |  |
| **TESTS ON FAECES** |  |  |  |  |
| Faecal occult blood |  |  |  |  |
| Faecal calprotectin |  |  |  |  |
| **OTHER TESTS WE HAVE NOT LISTED HERE** |  |  |  |  |

*Select one answer each row*

Q4a Frequency of POCT usage *ASK IF CODE 1 OR 3 IS SELECTED AT ONE ROW AT Q4*

Below is a list of point of care tests (POCTS) you indicated that you would use or currently use in your practice. Please tell us how often you would use or do use these

Please select the answer that best matches your views

|  | More than once per day | Daily | Weekly | Monthly | Once per year or less |
| --- | --- | --- | --- | --- | --- |
| **TESTS ON BLOOD** |  |  |  |  |  |
| **Cardiovascular** |  |  |  |  |  |
| Creatinine |  |  |  |  |  |
| Potassium |  |  |  |  |  |
| Sodium |  |  |  |  |  |
| Total cholesterol |  |  |  |  |  |
| HDL/LDL cholesterols |  |  |  |  |  |
| Triglycerides |  |  |  |  |  |
| Calcium |  |  |  |  |  |
| Uric Acid |  |  |  |  |  |
| BNP (B-natriuretic peptide) |  |  |  |  |  |
| D-dimer |  |  |  |  |  |
| Troponin |  |  |  |  |  |
| **Endocrine** |  |  |  |  |  |
| Blood glucose |  |  |  |  |  |
| HbA1c |  |  |  |  |  |
| TSH (thyroid stimulating hormone) |  |  |  |  |  |
| Free T4 or T3 |  |  |  |  |  |
| **Haematology** |  |  |  |  |  |
| INR |  |  |  |  |  |
| Haemoglobin |  |  |  |  |  |
| White cell count |  |  |  |  |  |
| Platelet count |  |  |  |  |  |
| Prothrombin time |  |  |  |  |  |
| **Infection related** |  |  |  |  |  |
| CRP (C-reactive protein) |  |  |  |  |  |
| Procalcitonin |  |  |  |  |  |
| HIV blood test |  |  |  |  |  |
| Hepatitis B |  |  |  |  |  |
| **Liver** |  |  |  |  |  |
| AST/ALT |  |  |  |  |  |
| Alkaline phosphatase |  |  |  |  |  |
| Bilirubin |  |  |  |  |  |
| Gamma GT (ɣ-glutamyltransferase) |  |  |  |  |  |
| Albumin |  |  |  |  |  |
| **Other (blood)** |  |  |  |  |  |
| ESR (Erythrocyte sedimentation rate) |  |  |  |  |  |
| CA125 |  |  |  |  |  |
| PSA (Prostate Specific Antigen) |  |  |  |  |  |
| Vitamin D |  |  |  |  |  |
| Vitamin B12 |  |  |  |  |  |
| Folate |  |  |  |  |  |
| Quantitative Beta HCG (Human chorionic gonadotropin) |  |  |  |  |  |
| Rheumatoid factor |  |  |  |  |  |
| ANA (anti-nuclear antibodies) |  |  |  |  |  |

|  | More than once per day | Daily | Weekly | Monthly | Once per year or less |
| --- | --- | --- | --- | --- | --- |
| **RESPIRATORY SAMPLES** |  |  |  |  |  |
| Throat swab for Group A Streptococci |  |  |  |  |  |
| Nasal swab for MRSA |  |  |  |  |  |
| Nose/throat swab for influenza |  |  |  |  |  |
| **TESTS ON URINE OR GENITAL FLUIDS** |  |  |  |  |  |
| Urine pregnancy test |  |  |  |  |  |
| Urine leucocytes or nitrite |  |  |  |  |  |
| Chlamydia |  |  |  |  |  |
| Gonorrhoea |  |  |  |  |  |
| Urine albumin:creatinine ratio |  |  |  |  |  |
| Urine total protein |  |  |  |  |  |
| Urine protein:creatinine ratio |  |  |  |  |  |
| **TESTS ON FAECES** |  |  |  |  |  |
| Faecal occult blood |  |  |  |  |  |
| Faecal calprotectin |  |  |  |  |  |
| **OTHER TESTS WE HAVE NOT LISTED HERE** |  |  |  |  |  |

*DISPLAY ANSWERS WHERE CODE 1 OR 3 WAS SELECTED AT Q4*

**Q5 Impact of Health Policy**

Do you think current changes in health care or policy are likely to have any impact on the use of POCTs? If so, please explain.

*Open end*

**Q6 Other comments**

Please share any other comments, including benefits and concerns about POCTs.

*Open end. Non-Mandatory*

**Finally we have a few questions about you**

**Q7** How many miles to your nearest emergency department that admits patients to hospital?

*Numeric. Range =0-150*

**Q8 Gender**

Please select your gender:

- Male
- Female

**Q9 Length of time for blood test**

How long does it typically take you to get results from a routine blood test, such as a full blood count?

- 1 day or more: -------------- days
- Less than 1 day: -------------- hours
- I already use a POCT for this test, so it is done immediately

**Q10 Year of qualification**

What year did you qualify as a doctor?

*Drop down list. Range 1960-2011*

**Q11 Patients in practice**

Approximately how many patients are registered in your practice?

*Numeric box. Range 0-20000; 0dp*

**Q12GP role**

Which of the following best describes your role in the practice?

- GP Partner/Principal
- Salaried GP
- Retainer GP
- Sessional GP
- GP Registrar/In training
- Locum GP
- Other (please specify) *Other specify*

**Q13 Practice location**

Is your practice based in a…

- Rural area
- Semi-rural area
- Urban area
- Suburban area

**Q14Hours worked**

How many hours per week do you work (on average)

*Numeric box. Range 0-60; 0dp*

**Thank you very much for your help!**

# Supplementary Table 1: Modified International Classification of Primary Care Codes

| **ICPC-2 Code** | **ICPC-2 Name** | **Condition** |
| --- | --- | --- |
| A01 | Pain, general/multiple sites (including chronic general pain, multiple aches) | Joint pain |
| A03 | Pyrexia of unknown origin (*NOT Glandular fever, which has its own category) | Fever |
| A04 | Weakness/tiredness, general (including chronic fatigue syndrome, exhaustion, fatigue, lassitude, lethargy, postviral fatigue) | tiredness |
| A10 | Bleeding/haemorrhage not otherwise specified | Haematological NOS |
| A70 | Tuberculosis (including tuberculosis infection of any body site, late effect of tuberculosis) | Tuberculosis |
| A71 | Measles (including complications of measles) | Measles |
| A72 / S70 | Chickenpox (including complications of chickenpox) / Herpes zoster (including post-herpetic neuralgia, shingles, herpes zoster ophthalmicus) | Chickenpox, Herpes zoster |
| A73 | Malaria | Malaria |
| A75/A77 | Infectious mononucleosis (including glandular fever, *M.pfeiffer*); Viral disease, other/not otherwise specified (including adenovirus, Coxsackie disease, dengue fever, Ross River fever) | Infectious mononucleosis, Viral disease other/NOS |
| A78.1 | Infectious disease, other/not otherwise specified (including brucellosis, infection unspecified site, Lyme disease, mycoplasma, Q fever, rickettsial disease, scarlet fever, sexually transmitted disease not otherwise specified, thrush not otherwise specified, toxoplasmosis); and gonorrhoea (male and female) and chlamydia (male and female) (X71/Y71) | STDs |
| A78.2 | Infectious disease, other/not otherwise specified (including brucellosis, infection unspecified site, Lyme disease, mycoplasma, Q fever, rickettsial disease, scarlet fever, sexually transmitted disease not otherwise specified, thrush not otherwise specified, toxoplasmosis) | Acute infection (bacterial versus viral NOS |
| A78.3 | Infectious disease, other/not otherwise specified (including brucellosis, infection unspecified site, Lyme disease, mycoplasma, Q fever, rickettsial disease, scarlet fever, sexually transmitted disease not otherwise specified, thrush not otherwise specified, toxoplasmosis) | Sepsis |
| A91/T87 | Abnormal result investigation not otherwise specified (including abnormal unexplained pathology/imaging test, electrolyte disorder, hyperglycaemia) | Electrolyte imbalances |
| A92 | Allergy/allergic reaction not otherwise specified (including allergic oedema, anaphylactic shock, angioneurotic oedema, food allergy) | Allergy/allergic reaction NOS |
| B78/80/81/82 | Hereditary haemolytic anaemia/Iron deficiency anaemia/Anaemia, vitamin B12-folate deficiency/Anaemia other, unspecified | Anaemia |
| B90 | HIV infection AIDS | HIV/AIDS |
| B99 | Blood/lymph/spleen disease, other (including complement defect, hypersplenism, immunodeficiency disorder, other/unspecified haematological abnormality, raise ESR, red cell abnormality, sarcoidosis, secondary polycythaemia) | Blood/lymph/spleen disease, other |
| D01/D02/D06 | Abdominal pain/cramps, general (including abdominal colic, abdominal cramps/discomfort/pan not otherwise specified, infant colic); Abdominal pain, epigastric (including epigastric discomfort, fullness, stomach ache/pain); Abdominal pain, localized, other (including colonic pain) | Abdominal pain |
| D16 | Rectal bleeding | Rectal bleeding |
| D70 | Gastrointestinal infection (including gastrointestinal infection/dysentery with specified organisms including campylobacter, giardia, salmonella, shigella, typhoid, cholera) | Gastrointestinal infection |
| D72 | Viral hepatitis (including all hepatitis presumed viral, chronic active hepatitis) | Viral hepatitis |
| D73 | Gastroenteritis, presumed infection (including diarrhoea/vomiting presumed to be infective, dysentery not otherwise specified, food poisoning, gastric flu) | Gastroenteritis |
| D86/D87 | Peptic ulcer, other (including gastric/gastrojejunal /marginal ulcer, acute erosion, Zollinger-Ellison syndrome); Stomach function disorder (including acute dilation stomach, duodenitis, gastritis) | Peptic ulcer |
| D88 | Appendicitis (including appendix abscess/perforation) | Appendicitis |
| D92 | Diverticular disease (including diverticulitis/diverticulosis of intestine) | Diverticular disease |
| D93/D94.0 | Irritable bowel syndrome (including mucous colitis, spastic colon), Chronic enteritis/ulcerative colitis (including Crohn's disease, endoscopic/imaging/histological findings) | Irritable bowel syndrome |
| D93/D94.1 | Irritable bowel syndrome (including mucous colitis, spastic colon), Chronic enteritis/ulcerative colitis (including Crohn's disease, endoscopic/imaging/histological findings) | Inflammatory bowel syndrome (including Crohn's and ulcerative colitis) |
| D97 | Liver Disease not otherwise specified (including liver failure, alcohol hepatitis, cirrhosis, hepatitis not otherwise specified, portal hypertension) | Liver Disease NOS |
| D98 | Cholecystitis/cholelithiasis (including biliary colic, cholangitis, gallstones) | Cholecystitis/cholelithiasias |
| D99.0 | Disease digestive system, other (including abnormal adhesions, coeliac disease, dumping syndrome, food intolerance, allergic/toxic/dietetic gastroenteropathy, ileus, intestinal obstruction, intussusception, lactose intolerance, malabsorption syndrome, mesenteric vascular disease, pancreatic disease, peritonitis, secondary megacolon, sprue) | Coeliac disease + Digestive disease NOS |
| D99.1 | Disease digestive system, other (including abnormal adhesions, coeliac disease, dumping syndrome, food intolerance, allergic/toxic/dietetic gastroenteropathy, ileus, intestinal obstruction, intussusception, lactose intolerance, malabsorption syndrome, mesenteric vascular disease, pancreatic disease, peritonitis, secondary megacolon, sprue) | Pancreatitis |
| F71/F79/F83/F93/F99 | Conjunctivitis, allergic (including allergic conjunctivitis with/without rhinorrhea) | Eye problems |
| H70/H71/H72 | Acute otitis media/myringitis (including acute suppurative otitis media, otitis media not otherwise specified, acute mastoiditis, acute tympanitis); Serous otitis media (including glue ear, otitis media with effusion (OME) | Otitis Media |
| H86 | Deafness (including congenital deafness, deafness on ear, partial/complete deafness both ears) and ear problems not otherwise specified (H82) | Deafness and Ear Problem NOS |
| K70 | Infection of circulatory system (including acute/subacute endocarditis, bacterial endocarditis, myocarditis, pericarditis (other than rheumatic) | Infection of circulatory system |
| K74/K75/K76 | Acute coronary syndrome / myocardial infarction /Ischaemic heart disease / angina / Cardiac disease, cardiac disease not otherwise specified | Acute Cardiac Disease |
| K77 | Heart failure (including cardiac asthma, congestive heart failure, heart failure not otherwise specified, left ventricular failure, pulmonary oedema, right ventricular failure) | Heart failure |
| K80 | Cardiac arrhythmia not otherwise specified (including atrial/junctional/ventricular premature beats, bradycardia, bigeminy, ectopic beats, extrasystoles, premature beats, sick sinus syndrome, ventricular fibrillation/flutter) | Cardiac Arrhythmia |
| K86/K87/K88 | Hypertension, uncomplicated (including essential hypertension, hypertension not otherwise specified, idiopathic hypertension); Hypertension, complicated (including malignant hypertension) | Hypertension |
| K90 | Stroke (including apoplexy, cerebral embolism/infarction/thrombosis/occlusion/stenosis/haemorrhage, cerebrovascular accident (CVA), subarachnoid haemorrhage) | Stroke |
| K93/K94 | Pulmonary embolism (including pulmonary (artery/vein) infarction, thromboembolism, thrombosis); Phlebitis/thrombophlebitis (including superficial/deep vein thrombosis, phlebothrombosis, portal thrombosis) | Pulmonary embolism / Deep Vein Thrombosis (DVT) |
| K99 | Cardiovascular disease, other (including aortic aneurism, arteriovenous fistula, arteritis, lymphoedema, oesophageal varices, other aneurysm, polyarteritis nodosa, vasculitis, varicose veins of sites other than lower extremities) | Cardiovascular disease, other |
| L02 | Back symptom/complaint (including backache not otherwise specified, thoracic back pain); Low back symptom/complaint (including lumbar/sacroiliac), coccydynia, lumbago, lumbalgia) | Back pain |
| L18 | Muscle pain (including fibromyalgia, fibrositis, myalgia, panniculitis, rheumatism) | Muscle pain |
| L70 | Infection of musculoskeletal system (including infective tenosynovitis, osteomyelitis, pyogenic arthritis) | Infection of bone/joint |
| L76/L95 | Fracture: other and Osteoporosis | Fractures and osteoporosis |
| L88/L89/L90/L99.0 | Musculoskeletal inflammation (including rheumatic disease) | Musculoskeletal inflammation (including rheumatic disease) |
| L88/L89/L90/L91.1 | Rheumatoid arthritis Drug Monitoring | RA/OA Drug monitoring |
| N71 | Meningitis/encephalitis | Meningitis/encephalitis |
| N89/N90/N95 | Migraine (including vascular headache with/without aura); Cluster headache; Tension headache | Migraine, Cluster headache, Tension headache |
| N93 | Carpal Tunnel Syndrome (including loss/impairment of superficial sensation affecting the thumb, index and middle finger, that may or may not split the ring finger. Dysaesthesia and pain worsen usually during the night, and may radiate to the forearm) | Carpal Tunnel Syndrome |
| N99 | Neurological disease, other (including cerebral palsy, dystonia, motor neuron disease, myasthenia gravis, neuralgia not otherwise specified) also including abnormal involuntary movements (N08), vertigo/dizziness (N17), head injury other (N80), multiple sclerosis (N86), epilepsy (N88) | Neurological disease, NOS |
| P06 | Sleep disturbance (including insomnia, nightmares, sleep apnoea, sleepwalking, somnolence), also including abnormal involuntary movements (N08), vertigo/dizziness (N17) | Sleep disturbance |
| P15/P16 | Chronic alcohol abuse (including alcohol brain syndrome, alcohol psychosis, alcoholism, delirium tremens); Acute alcohol abuse (including drunk) | Alcohol abuse |
| P17 | Tobacco abuse (including smoking problem) | Tobacco abuse |
| P19 | Drug abuse | Drug abuse |
| P70 | Dementia (including Alzheimer's disease, senile dementia) | Dementia |
| P73 | Affective psychosis (including bipolar disorder, hypomania, mania, manic depression) | Affective psychosis, lithium monitoring |
| P99 | Psychological disorder, other (including autism, neurosis not otherwise specified), and also schizophrenia (P72), depression (P76) suicide/suicide attempt (P77), post-traumatic stress disorder (P82) | Mental Health NOS |
| R02 | Shortness of breath/dyspnoea (including orthopnoea) | Shortness of breath |
| R05/R78 | Acute bronchitis/bronchiolitis (including chest infection, acute lower respiratory infection not otherwise specified, bronchitis not otherwise specified, chest infection not otherwise specified, laryngotracheobronchitis, tracheobronchitis); Cough; Pneumonia (R81), Pleurisy/pleural infusion (R82) | Chest infection / cough / LRTI |
| R71 | Whooping cough (including parapertussis, pertussis) | Whooping cough |
| R72 | Strep throat (including proven streptococcal pharyngitis/tonsilitis); also including R76/R90 | Strep throat / tonsillitis |
| R74 | Upper respiratory tract infection, acute (including acute rhinitis, coryza, head cold, nasopharyngitis, pharyngitis, URTI/URI) | bacterial infection URTI |
| R75 | Sinusitis acute/chronic (including sinusitis affecting any paranasal sinus) | Sinusitis |
| R80 | Influenza (including influenza-like illness, para-influenza) | Influenza |
| R83 | Respiratory infection, other (including chronic nasopharyngitis, chronic pharyngitis, chronic rhinitis not otherwise specified, diptheria, empyema, epiglottis, fungal respiratory infection, lung abscess, protozoal infection (without pneumonia) | Respiratory infection, other |
| R95/R96 | Chronic Obstructive Pulmonary Disease (including chronic obstructive airways (COAD), lung (COLD), pulmonary (COPD disease, chronic airways limitation (CAL), emphysema; Asthma (including reactive airways disease, wheezy bronchitis) | COPD/Asthma |
| R98 | Hyperventilation syndrome (including symptoms related to hyperventilation and relieved by rebreathing expired air) | Hyperventilation syndrome |
| R99 | Respiratory disease, other (including aspiration pneumonia, bronchiectasis, deviated nasal septum, lung complication of other disease, mediastinal disease, nasal polyp, other disease of larynx; pneumoconiosis, pneumothorax, pneumonitis due to allergy/chemicals/dust/fumes/mould, pulmonary collapse, respiratory failure) | Respiratory disease, other |
| S11 | Skin infection, post-traumatic (including infected post-traumatic wound/bite), including skin infection, other (S76) and impetigo (S84) | Skin infection |
| S20 | Corn/callosity | Corn/callosity |
| S72 | Scabies/other acariasis | Scabies |
| S74 | Dermatophytosis (including fungal skin infection, onychomycosis, pityriasis, versicolor, ringworm, tinea); also including infected finger/toe | Dermatophytosis |
| S77 | Malignant neoplasm of skin (including basal cell carcinoma, malignant carcinoma, rodent ulcer, squamous cell carcinoma of skin); also including moles (S82) | Malignant neoplasm of skin |
| S99 | Skin disease, other (including dermatitis artefacta, discoid lupus erythematosus, erythema multiforme, erythema nodosum, folliculitis, granuloma, granuloma, granuloma annulare, hyperkeratosis not otherwise specified, keloid, keratoacanthoma, lichen planus, neurodermatitis, onychogryphosis, rosacea, pigmentation, rhinophyma, scar, seborrhoeic or senile warts, striae atrophicae, vitiligo); also including rash (S06) and bruise (S16) and chronic skin ulcer (S97) and dermatitis (S87) | Skin disease, other |
| T11 | Dehydration (including water depletion) | Dehydration |
| T81/T85/T86 | Goitre (including non-toxic goitre, thyroid nodule)/Hyperthyroidism/thyrotoxicosis (including Grave's disease, toxic goitre)/Hypothyroidism/myxoedema | Hyper/hypothyroidism |
| T89/T90.0 | Diabetes insulin dependent/ Diabetes, non-insulin dependent (HbA1c testing) | Diabetes (HbA1c) |
| T89/T90.1 | Diabetes (glucose) | Diabetes (glucose) |
| T89/T90.2 | Diabetes (diabetic ketoacidosis) | Diabetes (diabetic ketoacidosis) |
| T89/T90.3 | Diabetes (urine test) | Diabetes (urine test) |
| T89/T90.4 | Diabetes (albumin creatinine ratio) | Diabetes (albumin creatinine ratio) |
| T89/T90.5 | Diabetes (not otherwise specified) | Diabetes (not otherwise specified) |
| T91 | Vitamin/nutritional deficiency (including beri-beri, dietary mineral deficiency, iron deficiency without anaemia, malnutrition, marasmus, scurvy) | Vitamin/nutritional deficiency |
| T92 | Gout | gout |
| T93 | Lipid disorder (including abnormality of lipoprotein level, hyperlipidaemia, raised level of cholesterol/triglycerides, xanthoma) | Lipid disorder |
| T99 | Endocrine/metabolic/nutritional disease, other (including acromegaly, adrenal/ovarian/pituitary/parathyroid/testicular/other endocrine dysfunction, amyloidosis, crystal arthropathy, Cushing's syndrome, cystic fibrosis, diabetes insipidus, Gilbert's syndrome, hyperaldosteronism, osteomalacia, porphyria, precocious/delayed puberty, pseudo-gout, renal glycosuria, thyroiditis) | Endocrine/metabolic/nutritional disease, other |
| U06 | Haematuria (including blood in urine) | Haematuria |
| U14 | Kidney symptom/complaint (including kidney pain, kidney trouble, renal colic); and Urinary calculus (U95) | Renal colic |
| U28/U99 | Urinary disease, other (including bladder diverticulum, hydronephrosis, hypertrophic kidney, obstruction bladder neck, renal failure, urethral caruncle, urethral stricture, ureteric reflux, uraemia) | Acute and Chronic Renal Impairment / failure |
| U70/U71 | Pyelonephritis/pyelitis (including infection of kidney, renal/perinephric abscess) / Cystitis/urinary infection, other (including lower urinary tract infection, urinary tract infection not otherwise specified) and Dysuria | Urinary tract infection |
| U88 | Glomerulonephritis/nephrosis (including acute glomerulonephritis, analgesic nephropathy, chronic glomerulonephritis, nephritis, nephropathy, nephrosclerosis, nephrotic syndrome) | Glomerulonephritis/nephrosis |
| WO3 | Antepartum bleeding | Antepartum bleeding |
| W05 (+D09/D10, D11) | Pregnancy vomiting/nausea (including hyperemesis, morning sickness in confirmed pregnancy) | Nausea, vomiting |
| W15/Y10 | Infertility/subfertility, female (including sterility, primary and secondary); Infertility, male (including failure of conception after 2 years of trying) | Infertility |
| W80 | Ectopic Pregnancy | Ectopic Pregnancy |
| W81 | Toxaemia of pregnancy (including eclampsia, hypertension, oedema and proteinuria in pregnancy, pre-eclampsia) | Toxaemia of pregnancy |
| W82 | Abortion, spontaneous (including abortion threatened/complete/incomplete/missed/habitual, miscarriage) and disorder of pregnancy (W99) | Abortion, spontaneous |
| X06/X08 | Menstruation excessive (including menorrhagia, pubertal bleeding); Intermenstrual bleeding (including breakthrough bleeding, dysfunctional uterine bleeding, metrorrhagia, ovulation bleeding, spotting) | Menstruation excessive |
| X11 | Menopausal symptom/complaint (including atrophic vaginitis, menopause syndrome, symptom/complaint related to menopause, senile vaginitis) | Menopausal symptom/complaint |
| X14 | Vaginal discharge (including fluor vaginalis, leukorrhoea), and genital candidiasis (X72) and vaginosis (X84) | Vaginal discharge |
| X21 | Breast symptom/complaint female, other (including mastitis (non-lactating), mastopathy, galactorrhoea) | Breast symptom/complaint female, other |
| X99/Y99 | Genital disease, female, other (including Bartholin cyst/abscess, endometriosis, genital tract fistula female, pelvic congestion syndrome, physiological ovarian cyst) Genital disease, male, other (including other disease of male breast, epididymal cyst, spermatocele, torsion of the testis) | Genital disease, female, other; genital disease, male, other |
| XX00 (not ICPC code) | INR / anticoagulation | INR / anticoagulation |
| XX01 (not ICPC code) | Rare endocrine disorders | Rare endocrine disorders |
| XX02 (not ICPC code) | Urea and Electrolytes | Urea and Electrolytes |
| XX03 (not ICPC code) | Dysphagia | Dysphagia |
| XX04 (not ICPC code) | Neutropenia | Neutropenia |
| XX05 (not ICPC code) | Hypoxia | Hypoxia |
| XX06 (not ICPC code) | Arterial/Venous Ulcer | Arterial/Venous Ulcer |
| XX07 (not ICPC code) | Cancer (All) | Cancer |
| XX08 (not ICPC code) | Pregnancy | Pregnancy |
| XXX (not ICPC code) | Uncodable (because it is a test for several conditions, or is ambiguous) | Uncodable |
| XXX.0 (not ICPC code) | OTHER | OTHER |
| Y06 | Prostate symptom/complaint, other (including prostatism) | Prostate symptom/complaint, other |
| Y29 | Genital symptom/complaint male, other | Genital symptom/complaint male, other |

# Supplementary Table 2: Details of diabetes mellitus related conditions/testing recorded by respondents and coded by the Modified International Classification of Primary Care Codes (Supplementary Table 1) for the three testing scenarios. Conditions/classifications marked with asterisks appear in the top 20 (Tables 2 – 4)

|  | **Diagnosis** |  | **Referral reduction** | | **Monitoring** |  |
| --- | --- | --- | --- | --- | --- | --- |
| **DM related condition/classifications** | **Frequency** | **Percentage of total recorded** | **Frequency** | **Percentage of total recorded** | **Frequency** | **Percentage of total recorded** |
| Diabetes not otherwise specified (NOS) | *387 | 69.7 | *133 | 76.4 | *527 | 80.8 |
| Diabetes (HbA1c) | 25 | 4.5 | 3 | 1.7 | *73 | 11.2 |
| Diabetes (glucose) | *98 | 17.7 | 17 | 9.8 | *37 | 5.7 |
| Diabetes (DKA) | 30 | 5.4 | 18 | 10.3 | 3 | 0.5 |
| Diabetes (urine) | 13 | 2.3 | 3 | 1.7 | 11 | 1.7 |
| Diabetes (ACR) | 2 | 0.4 |  |  | 1 | 0.2 |
| **Total recorded** | **555** |  | **174** |  | **652** |  |

# Supplementary Table 3: Sub classification of cancers recorded by respondents and coded under the Cancer (all) category for the three testing scenarios (does not include skin cancers, which have their own ICPC-2 code)

|  | **Diagnosis** |  | **Referral reduction** | | **Monitoring** |  |
| --- | --- | --- | --- | --- | --- | --- |
| **Cancer Type** | **Frequency** | **Percentage of total recorded** | **Frequency** | **Percentage of total recorded** | **Frequency** | **Percentage of total recorded** |
| Cancer not otherwise specified (NOS) | 20 | 23.5 | 16 | 22.9 | 21 | 21.0 |
| Urological cancers | 36 | 42.4 | 27 | 38.6 | 58 | 58.0 |
| Gastrointestinal cancers | 12 | 14.1 | 9 | 12.9 | 8 | 8.0 |
| Gynaecological cancers | 7 | 8.2 | 11 | 15.7 | 7 | 7.0 |
| Breast cancer | 2 | 2.4 | 5 | 7.1 | 1 | 1.0 |
| Lung cancer | 4 | 4.7 | 1 | 1.4 | 3 | 3.0 |
| Cancers of the brain | 1 | 1.2 |  |  |  |  |
| Endocrine cancers | 1 | 1.2 | 1 | 1.4 | 1 | 1.0 |
| Cancers of the blood | 2 | 2.4 |  |  | 1 | 1.0 |
| **Total recorded** | **85** |  | **70** |  | **100** |  |

# Supplementary Table 4: Potential correlations between demographic data and number of conditions listed by respondents

|  | Time to blood test results | Distance of practice from nearest ED | Practice Size |
| --- | --- | --- | --- |
| Diagnosis | χ^2^(5) = 5.557, *p* = 0.352 | χ^2^(5) = 10.265, *p* = 0.068 | χ^2^(5) = 10.833, *p* = 0.055 |
| Referrals | χ^2^(5) = 4.058, *p* = 0.541 | χ^2^(5) = 9.294, *p* = 0.098 | χ^2^(5) = 5.116, *p* = 0.402 |
| Monitoring | χ^2^(5) = 4.365, *p* = 0.498 | χ^2^(5) = 9.784, *p* = 0.082 | χ^2^(5) = 8.623, *p* = 0.125 |
